# Specimen origin, type and testing laboratory are linked to longer turnaround times for HIV viral load testing in Malawi

**DOI:** 10.1371/journal.pone.0173009

**Published:** 2017-02-24

**Authors:** Peter A. Minchella, Geoffrey Chipungu, Andrea A. Kim, Abdoulaye Sarr, Hammad Ali, Reuben Mwenda, John N. Nkengasong, Daniel Singer

**Affiliations:** 1 Laboratory Leadership Service assigned to Division of Global HIV and Tuberculosis, Centers for Disease Control and Prevention, Atlanta, Georgia, United States of America; 2 Division of Global HIV and Tuberculosis, Centers for Disease Control and Prevention, Lilongwe, Malawi; 3 Division of Global HIV and Tuberculosis, Centers for Disease Control and Prevention, Atlanta, Georgia, United States of America; 4 Ministry of Health, Lilongwe, Malawi; Hôpital Bichat-Claude Bernard, FRANCE

## Abstract

**Background:**

Efforts to reach UNAIDS’ treatment and viral suppression targets have increased demand for viral load (VL) testing and strained existing laboratory networks, affecting turnaround time. Longer VL turnaround times delay both initiation of formal adherence counseling and switches to second-line therapy for persons failing treatment and contribute to poorer health outcomes.

**Methods:**

We utilized descriptive statistics and logistic regression to analyze VL testing data collected in Malawi between January 2013 and March 2016. The primary outcomes assessed were greater-than-median pretest phase turnaround time (days elapsed from specimen collection to receipt at the laboratory) and greater-than-median test phase turnaround time (days from receipt to testing).

**Results:**

The median number of days between specimen collection and testing increased 3-fold between 2013 (8 days, interquartile range (IQR) = 6–16) and 2015 (24, IQR = 13–39) (*p<0*.*001)*. Multivariable analysis indicated that the odds of longer pretest phase turnaround time were significantly higher for specimen collection districts without laboratories capable of conducting viral load tests (adjusted odds ratio (aOR) = 5.16; 95% confidence interval (CI) = 5.04–5.27) as well as for Malawi’s Northern and Southern regions. Longer test phase turnaround time was significantly associated with use of dried blood spots instead of plasma (aOR = 2.30; 95% CI = 2.23–2.37) and for certain testing months and testing laboratories.

**Conclusion:**

Increased turnaround time for VL testing appeared to be driven in part by categorical factors specific to the phase of turnaround time assessed. Given the implications of longer turnaround time and the global effort to scale up VL testing, addressing these factors via increasing efficiencies, improving quality management systems and generally strengthening the VL spectrum should be considered essential components of controlling the HIV epidemic.

## Introduction

In an effort to control the HIV epidemic and meet the ambitious “90-90-90” targets set forth by the Joint United Nations Programme on HIV/AIDS (UNAIDS) in 2014 [1], low and middle income countries (LMICs) have rapidly expanded the number of individuals on antiretroviral therapy (ART). Globally, the number increased by an estimated 2 million to more than 17 million people on ART between the end of 2014 and the end of 2015 [2]. Such regional increases in ART coverage have intensified the need for scale-up of ART monitoring, for which the current gold standard and World Health Organization (WHO) recommendation [3, 4], is viral load (VL) testing.

Treatment monitoring via VL testing is the standard of care in developed countries and is preferred over its predecessor, immunological monitoring, because it enables earlier and more accurate detection of treatment failure [5, 6]. However, a 2012/2013 World Health Organization WHO survey of LMICs indicated that just 50% of individuals on ART have access to VL monitoring [7], and in many countries there are significant barriers to scaling-up VL testing. Among the most prominent of those barriers are the cost and complexity of VL testing [8], which limits the number of testing sites and trained technicians in resource-constrained settings [9, 10] and leaves aspects of testing, such as turnaround time, vulnerable [11]. Longer turnaround time delays initiation of treatment adherence counseling and/or switch to second-line ART in patients experiencing treatment failure. These delays negate an advantage of VL testing over immunological monitoring and lead to poorer health outcomes including increased risk of opportunistic infections [12], prolonged immune activation [13], development of drug resistance [14], and increased mortality [15].

Given the links between delayed switch to second-line ART and poor health outcomes, improved understanding of factors that contribute to longer VL turnaround time, which may cause such delays, is needed. This is particularly important in the context of viral load scale-up [16], which may exacerbate the effects of such factors. Thus, this study examined viral load turnaround time and factors that affected turnaround time using nationally representative VL testing data from Malawi between 2013 and March 2016, a time period during which there was rapid scale-up of VL testing.

## Methods

### Setting

The data utilized in this study were extracted from the Malawi Laboratory Information Management system (LIMS), established in 2012 and designed to collect laboratory data on all HIV viral load and early infant diagnosis tests conducted in Malawi. The extraction period for this analysis was for VL tests conducted from January 1, 2013 to March 31, 2016.

VL testing in Malawi is initiated at ART clinics, where a VL laboratory requisition form is completed for each specimen collected. The form includes patient identifiers, demographic information, the type of specimen collected, and whether the requisition is for a routine or targeted VL test. Following completion of the form, the specimen is transported to one of Malawi’s nine molecular laboratories capable of conducting HIV VL testing. Upon receipt at the VL testing laboratory, data from the requisition form are entered into the LIMS and the specimen is placed in a testing queue. Following testing, the result is entered into the LIMS and a report is printed for delivery back to the referring clinic and patient. Data from the LIMS at the nine VL testing labs are routinely synced with a central server via dedicated internet connections. For the period during which data for this study were collected, Malawi’s VL testing guidelines were in line with WHO treatment recommendations [3, 4].

### Measures

**Fig 1:**
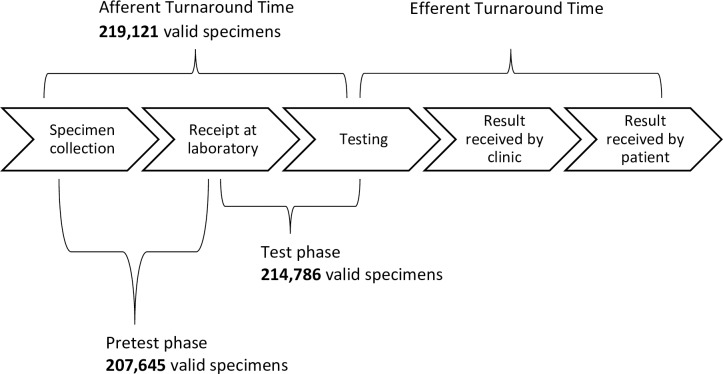
Turnaround time definitions and specimens used in turnaround time calculations. Afferent turnaround time is defined as the number of days elapsed between specimen collection and specimen testing. Two phases are defined within afferent turnaround time: Pretest phase, the number of days elapsed between specimen collection and date of receipt at the laboratory; and Test phase, the number of days elapsed between specimen receipt at the laboratory and the date of testing. Efferent turnaround time, which was not assessed in this study, is defined as the number of days elapsed between the date of testing and the date the result is received by the patient. Of 243,539 viral load specimens tested between 2013 and March 2016: 219,121 specimens had valid dates of both specimen collection and testing and thus were included in calculations of afferent turnaround time, 207,645 specimens had valid dates of both specimen collection and receipt at the laboratory and thus were included in calculations of pretest phase turnaround time, and 214,786 specimens had valid dates of both receipt and testing and thus were included in calculations for test phase turnaround time. Valid dates were defined as those that were present and plausible (e.g., an implausible testing date would be one that fell prior to the specimen collection date).

Afferent turnaround time was defined as the number of days elapsed between the date of specimen collection and the date of specimen testing (Fig 1). More precise measures of turnaround time within the afferent period were pretest phase turnaround time, which was defined as the number of days elapsed between specimen collection and receipt at the laboratory; and test phase turnaround time, which was defined as the number of days elapsed between receipt at the laboratory and specimen testing. Data on efferent turnaround time was not systematically collected in Malawi by LIMS or any other means, and thus not included in the analysis. Since Malawi’s LIMS collects data on all specimens undergoing VL testing, regardless of missing or implausible dates, it was necessary to distinguish between specimens with valid and invalid dates in order to accurately calculate turnaround time. Valid specimens were defined as those that had present and plausible collection, receipt, or testing dates depending on the turnaround time being calculated. All other specimens were excluded from turnaround time calculations. Overall medians for each phase of turnaround time were calculated and used as cut-offs to define longer turnaround times. The primary outcomes used in regression analyses were longer (i.e., greater-than-median) afferent, pretest phase and test phase turnaround time.

Factors included in the analyses were hypothesized *a priori* to be predictors of longer turnaround time. Since pretest phase and test phase turnaround times assess different parts of the VL spectrum, predictors differed by phase. For pretest phase, predictors were: region, presence of a molecular laboratory in the collection district, and collection month. Region is a geopolitical distinction defined by the Government of Malawi, presence of a molecular laboratory in the collection district identifies districts with and without laboratories capable of conducting viral load testing, and collection month is the month during which the specimen was collected. Analysis on collection month was conducted using only 2015 data in order to better understand recent temporal trends in turnaround time. Predictors for test phase were: sample type, testing laboratory, and receipt month. Sample type distinguishes whether the specimen was collected as a dried blood spot (DBS) or plasma, testing laboratory is a de-identified variable for the laboratory that conducted the VL test, and receipt month is the month during which the specimen was received at the testing laboratory. Similar to collection month, analyses on receipt month were restricted to 2015 data.

### Data analyses

Frequencies were generated for categorical variables and means, standard deviations, medians, and interquartile ranges (IQR) for normally distributed and non-normally distributed continuous variables, respectively. Wilcoxon-Mann-Whitney tests were used to compare specimen collection volumes and turnaround times.

To examine the relationship between turnaround time and the volume of specimens collected, a variable was created for all valid specimens that was equal to the total number of VL specimens collected nationally during the same month. That variable was standardized and assessed for univariate association with longer afferent turnaround time. A similar approach was utilized to assess the relationship between longer turnaround time and both the volume of specimens collected per district and the volume of tests conducted per testing laboratory. Univariate and multivariable logistic regression were employed to assess associations between phase-specific categorical factors and greater-than-median pretest phase and test phase turnaround times. Factors for both phases were assessed independently and also included in multivariable models. Associations with specimen collection and receipt months were restricted to data collected during 2015, only. All analyses were performed using SAS 9.3 (Cary, NC).

### Ethics

The protocol for this analysis was approved by the Centers for Disease Control and Prevention (CDC) Institutional Review Board (IRB) and the Malawi National Health Science Research Committee (NHSRC).

## Results

Monthly volumes of VL specimens collected increased significantly from 2013 (median: 2,171 tests, IQR = 1,557–3,481) to 2014 (median: 5,622 tests, IQR: 4,276–6,112; *p<0*.*001*) and from 2014 to 2015 (median: 10,296, IQR: 9,001–12,025; *p = 0*.*002*). This was paralleled by year-to-year increases in the number of clinics referring specimens for viral load testing and median afferent turnaround time. Overall, the vast majority (97.6%) of VL tests were conducted on specimens referred for routine testing, which had a higher median turnaround time (median: 21 days, IQR: 10–41) compared to specimens referred for targeted testing (median: 10 days, IQR: 6–19). Most specimens (86.1%) had a VL result that was less than or equal to 1000 copies/ml (Table 1).

**Table 1:** Test characteristics for viral load specimens tested, 2013-March 2016.

Year	Specimens tested	Clinics referring specimens for viral load testing	Laboratories conducting viral load testing	Median afferent TAT^a^, days (IQR) n = 219,121	DBS specimens tested, % n = 243,339	Routine tests, % n = 239,770	Viral Load Result ≤ 1000 copies/ml, % n = 243,539
2013	32,516	124	3	8 (6–16)	1,847, 5.7%	29,199, 93.2%	27,954, 86.0%
2014	61,579	268	6	14 (7–25)	25,325, 41.1%	58,844, 97.1%	52,061, 84.5%
2015	103,848	550	9	24 (13–39)	74,730, 72.0%	100,818, 98.4%	89,025, 85.7%
Jan 2016-Mar 2016	45,596	571	9	48 (34–67)	39,523, 86.7%	45,159, 99.5%	40,650, 89.2%
2013-Mar 2016	243,539	-	-	21 (10–41)	141,425, 58.1%	234,020, 97.6%	209,690, 86.1%

Values are reported as median (interquartile range) or n, %.

Specimens tested: all viral load specimens tested and recorded in the LIMS. Clinics referring specimens for viral load testing: the number of clinics in the given year that referred ≥1 specimen for viral load testing. Laboratories conducting viral load testing: the number of laboratories in the given year that conducted ≥1 viral load test. Median afferent TAT: median number of days from specimen collection date to specimen testing date, values calculated using only specimens with valid dates. DBS specimens: specimens collected as dried blood spots (vs. plasma). Routine tests: viral load specimens collected for routine (vs. targeted) testing. Virally suppressed: viral load result ≤ 1000 copies/ml.

Abbreviations: TAT, afferent turnaround time; IQR, Interquartile range; DBS, dried blood spot.

^a^ Values increased significantly from 2013 to 2014 (*p<0*.*001*), from 2014 to 2015 (*p<0*.*001*), and from 2015 to 2016 (*p<0*.*001*) Wilcoxon-Mann-Whitney test.

**Fig 2:**
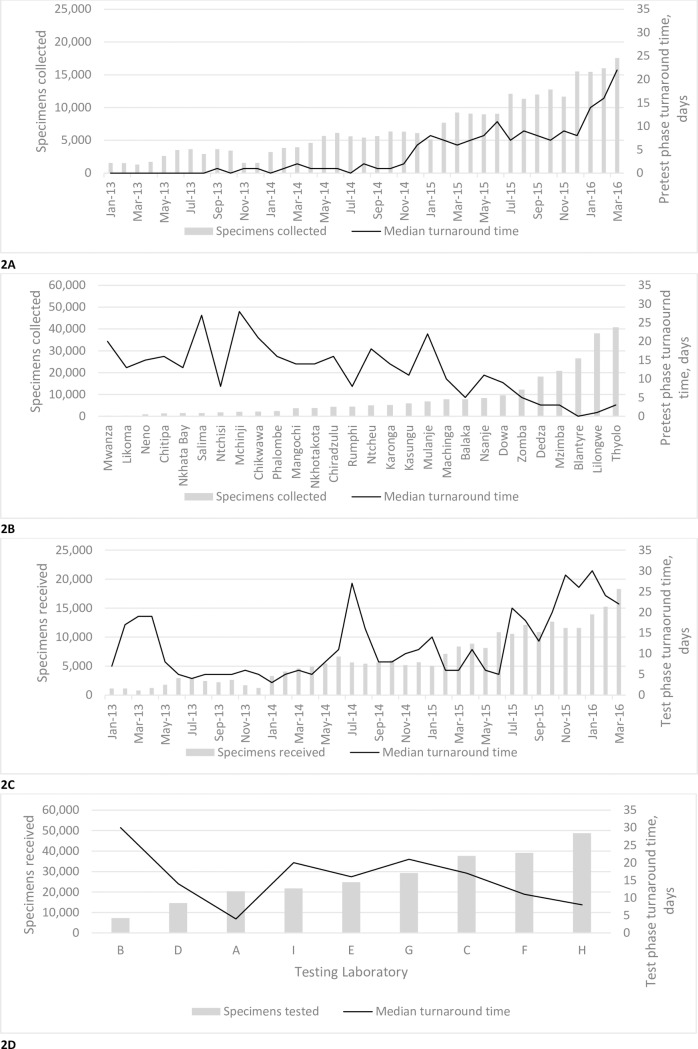
Volumes of viral load specimens collected/received/tested and corresponding median pretest phase and test phase turnaround times. 2(A) National number of specimens collected and referred for viral load testing by month and corresponding monthly median pretest phase turnaround time. 2(B) Number of specimens referred by district and corresponding median pretest phase turnaround time by district. 2(C) National number of specimens received at the laboratory for viral load testing by month and corresponding monthly median test phase turnaround time. 2(D) Number of specimens received per laboratory and corresponding median test phase turnaround time by laboratory. All pretest and test phase turnaround times were calculated using only specimens with valid dates.

Nationally, while greater volumes of viral load specimens collected per month were associated with longer afferent turnaround time (odds ratio (OR) = 2.65, 95% confidence interval (CI) = 2.64–2.68), greater volumes of specimens collected per district (OR = 0.45, 95% CI = 0.45–0.46) and greater volumes of specimens tested per laboratory (OR = 0.65, 95% CI = 0.65–0.66) were not. Fig 2 graphically shows more precise breakdowns of this relationship. In Fig 2A, median pretest phase turnaround times track monthly changes in national specimen collection volumes, but that is not the case for district specimen collection volumes arranged in ascending order (Fig 2B). Similarly, median test phase turnaround time trends alongside monthly changes in the national volume of specimens received (Fig 2C), but no such relationship is evident between test phase turnaround time and the volume of specimens received at laboratories (Fig 2D).

Categorical predictors of longer pretest phase turnaround time were assessed (Table 2). In univariate models, the odds of longer turnaround time were slightly increased for Malawi’s Northern and Southern regions compared to the Central region and substantially increased for districts without molecular laboratories compared to those with molecular laboratories. For 2015 specimen collection month, only February had significantly decreased odds of longer turnaround time compared to the reference month of January.

**Table 2:** Factors associated with longer pretest phase turnaround time for viral load specimens collected in Malawi, 2013-March 2016.

Factor		Specimens collected^a^	Median pretest phase TAT^b^ (IQR), days	Unadjusted OR^b^ (95% CI)	Adjusted OR^b^ (95% CI)
Region	Northern	33,477	7 (2–13)	1.05 (1.02–1.08), *p*<0.001	1.42 (1.38–1.47), *p*<0.001
	Central	85,840	6 (0–14)	ref	ref
	Southern	124,208	7 (0–16)	1.03 (1.01–1.05), *p* = 0.008	1.45 (1.42–1.49), *p*<0.001
Molecular Lab in Collection District	No	101,222	12 (6–21)	5.44 (5.34–5.55), *p*<0.001	5.16 (5.04–5.27), *p*<0.001
	Yes	142,317	2 (0–8)	ref	ref
2015 Collection Month	January	5,144	7 (1–13)	ref	ref
	February	7,691	6 (1–11)	0.71 (0.65–0.77), *p*<0.001	0.59 (0.54–0.64), *p*<0.001
	March	9,244	7 (3–12)	0.97 (0.90–1.05), *p* = 0.53	0.83 (0.77–0.90), *p*<0.001
	April	9,082	9 (3–16)	1.63 (1.51–1.75), *p*<0.001	1.45 (1.34–1.57), *p*<0.001
	May	8,957	14 (5–23)	2.34 (2.16–2.52), *p*<0.001	2.21 (2.04–2.40), *p*<0.001
	June	9,045	7 (2–13)	1.06 (0.98–1.14), *p* = 0.14	0.91 (0.84–0.98), *p* = 0.017
	July	12,099	11 (4–16)	1.84 (1.71–1.97), *p*<0.001	1.48 (1.37–1.60), *p*<0.001
	August	11,348	8 (2–15)	1.21 (1.13–1.30), *p*<0.001	0.94 (0.87–1.02), *p* = 0.5
	September	11,951	7 (1–15)	1.08 (1.00–1.16), *p* = 0.08	0.84 (0.78–0.90), *p*<0.001
	October	12,592	8 (3–16)	1.45 (1.35–1.56), *p*<0.001	1.23 (1.14–1.33), *p*<0.001
	November	11,420	12 (4–18)	2.07 (1.93–2.22), *p*<0.001	1.67 (1.55–1.81), *p*<0.001
	December	14,444	19 (8–29)	3.57 (3.32–3.84), *p*<0.001	2.58 (2.39–2.79), *p*<0.001

Logistic regression was utilized to model the relationship between longer pretest phase turnaround time and “Region”, “Molecular Lab in Collection District”, and “2015 Collection Month”. Referent categories were ‘Central Region’, districts with molecular labs (i.e., ‘Yes’), and ‘January’, respectively. In the adjusted models, the relationship between longer pretest phase turnaround time and a given factor was adjusted for the other two factors presented in the table (e.g., the adjusted model for region was adjusted for “Molecular Lab in Collection District” and “Specimen Collection Month”). ‘Specimen Collection Month’ as a factor in the adjusted model included both the month and year (e.g., September 2014). Longer pretest phase turnaround time was defined as greater-than-median pretest phase turnaround time.

Abbreviations: TAT, turnaround time; IQR, Interquartile range; OR, odds ratio; CI, confidence interval.

^a^ Specimens collected reflects the total number of specimens collected, regardless of validity. Note that specimens collected per 2015 collection month includes only specimens collected during 2015.

^b^ Specimens included in the turnaround time calculation and regression analyses for “Region” and “Molecular Lab in Collection District” (n = 207,638). Specimens included in the turnaround time calculation and regression analyses for “2015 Collection Month” (n = 112,524).

Adjusting for multiple predictors in the same model affected associations between longer pretest phase turnaround time and categorical factors. Compared to the univariate model, the odds of longer turnaround time for Malawi’s Northern and Southern regions increased in the adjusted model, while the odds of longer turnaround time for districts without molecular laboratories decreased slightly upon adjustment (Table 2). In the adjusted model for collection month, the odds of longer turnaround time were decreased for some months, but increased for others relative to the univariate model; reflecting inter-month variability in factors’ influence on turnaround time.

Categorical predictors of longer test phase turnaround time were also assessed (Table 3). The univariate odds of longer turnaround time for DBS were increased compared to plasma. For testing laboratories and 2015 receipt month, some laboratories and months had increased univariate odds of longer turnaround time while others had decreased odds compared to their respective reference categories. The months of July, October, November and December stood out with particularly high odds of longer test phase turnaround time.

**Table 3:** Factors associated with longer test phase turnaround time for viral load specimens received by testing laboratories in Malawi, 2013-March 2016.

Factor		Specimens received^a^	Median test phase TAT^b^ (IQR), days	Unadjusted OR^b^ (95% CI)	Adjusted OR^b^ (95% CI)
Sample Type	DBS	141,425	18 (8–30)	3.32 (3.26–3.39), *p*<0.001	2.30 (2.23–2.37), *p*<0.001
	Plasma	101,914	7 (3–18)	ref	ref
Testing Lab	A	20,370	4 (1–7)	0.30 (0.29–0.32), *p*<0.001	0.40 (0.37–0.42), *p*<0.001
	B	7,308	30 (15–50)	5.41 (5.02–5.82), *p*<0.001	4.03 (3.69–4.39), *p*<0.001
	C	37,594	11 (4–34)	1.36 (1.32–1.40), *p*<0.001	2.80 (2.70–2.91), *p*<0.001
	D	14,594	14 (8–24)	1.61 (1.55–1.68), *p*<0.001	1.27 (1.21–1.33), *p*<0.001
	E	24,847	16 (7–25)	1.98 (1.91–2.05), *p*<0.001	2.15 (2.06–2.24), *p*<0.001
	F	39,084	11 (5–20)	ref	ref
	G	29,258	21 (12–31)	3.61 (3.49–3.72), *p*<0.001	3.72 (3.57–3.87), *p*<0.001
	H	48,754	8 (5–17)	0.74 (0.72–0.76), *p*<0.001	0.57 (0.55–0.60), *p*<0.001
	I	21,730	20 (10–37)	3.37 (3.26–3.49), *p*<0.001	2.71 (2.60–2.82), *p*<0.001
2015 Receipt Month	January	5,049	8 (3–21)	ref	ref
	February	7,100	6 (4–13)	0.47 (0.43–0.51), *p*<0.001	0.31 (0.28–0.34), *p*<0.001
	March	8,362	9 (4–18)	0.87 (0.81–0.94), *p*<0.001	0.58 (0.54–0.63), *p*<0.001
	April	8,871	7 (5–13)	0.41 (0.37–0.44), *p*<0.001	0.23 (0.21–0.25), *p*<0.001
	May	8,132	10 (5–23)	1.15 (1.06–1.24), *p*<0.001	0.73 (0.67–0.80), *p*<0.001
	June	10,850	7 (3–25)	0.97 (0.90–1.04), p = 0.36	0.75 (0.69–0.81), *p*<0.001
	July	10,549	21 (13–27)	4.64 (4.31–4.99), *p*<0.001	3.39 (3.12–3.67), *p*<0.001
	August	12,095	12 (6–18)	1.17 (1.09–1.26), *p*<0.001	0.77 (0.71–0.83), *p*<0.001
	September	10,857	16 (9–30)	1.91 (1.78–2.06), *p*<0.001	1.35 (1.24–1.46), *p*<0.001
	October	12,566	31 (14–42)	4.95 (4.60–5.32), *p*<0.001	3.83 (3.54–4.16), *p*<0.001
	November	11,511	43 (22–52)	13.71 (12.60–14.91), *p*<0.001	11.40(10.38–12.51), *p*<0.001
	December	11,427	29 (20–47)	10.01 (9.24–10.85), *p*<0.001	6.22 (5.69–6.80), *p*<0.001

Logistic regression was utilized to model the relationship between longer test phase turnaround time and “Sample Type”, “Testing Lab”, and “2015 Receipt Month”. Referent categories were ‘Plasma’, ‘Laboratory F’, and ‘January’, respectively. Laboratory F served as the referent category because it had the highest number of specimens included in the analysis. In the adjusted models, the relationship between longer test phase turnaround time and a given factor was adjusted for the other two factors presented in the table (e.g., the adjusted model for “Sample Type” was adjusted for “Testing Lab” and “Specimen Receipt Month”). ‘Specimen Receipt Month’ as a factor in the adjusted model included both the month and year (e.g., September 2014). Longer test phase turnaround time was defined as greater-than-median test phase turnaround time.

Abbreviations: TAT, turnaround time; IQR, Interquartile range; OR, odds ratio; CI, confidence interval.

^a^ Specimens received reflects the total number of specimens received, regardless of validity. Note that the number of specimens received per 2015 receipt month includes only specimens received during 2015.

^b^ Specimens included in the turnaround time calculation and regression analyses for “Sample Type” and “Testing Lab” (n = 214,601). Specimens included in the turnaround time calculation and the regression analyses for “2015 Receipt Month” (n = 107,479).

In an adjusted model, the odds of longer turnaround time for DBS were decreased relative to the univariate model, though still significantly higher than for plasma. For testing laboratory, Table 3 shows that after adjustment, the odds of longer turnaround time increased for some laboratories, but decreased for others.

## Discussion

This study shows that increased turnaround time for VL testing appeared to be driven in part by categorical factors specific to the phase of turnaround time assessed. Indeed, factors, such as the presence of a molecular testing laboratory in the collection district, specimen type, and testing laboratory appeared to contribute to longer turnaround time; potentially putting patients at risk of poorer health outcomes by delaying adherence counseling and/or switch to second-line therapy. Shortening afferent turnaround time and ensuring that health outcomes are not affected by laboratory or operational delays will require identification of the specific causes responsible for longer turnaround time and implementation of measures to mitigate those causes in the future.

While turnaround times were lower in districts and laboratories that collected and received higher volumes of specimens (Fig 2B and 2D), suggesting factors such as familiarity with workflow shorten turnaround time, a similar relationship between increasing specimen volumes and shortened turnaround time was not evident on a national scale (Fig 2A and 2C). These data, along with the overall increase in median afferent turnaround time between 2013 and 2015, support the assertion that factors contributing to shorter turnaround time, were outweighed by factors contributing to longer turnaround time. Understanding what those factors are, how they influence turnaround time, and in which ways they can be addressed is an important step in the effort to shorten turnaround time.

HIV VL testing’s dependence on expensive equipment, dedicated laboratory space and highly trained technicians limits its accessibility for many ART patients in LMICs [9, 10]. The current preferred strategy to overcome this accessibility problem is through centralized testing and a robust sample transport network. Use of DBS instead of plasma, which in many cases requires neither a phlebotomist nor a cold-chain, further enhances the cost-effectiveness and feasibility of centralized testing. WHO recommends that DBS can be used effectively at a threshold of 1000 copies/ml in most laboratory settings [17], and its feasibility has been documented as part of a centralized testing network in Malawi [18]. However, impacts of centralized testing and use of DBS on aspects of the VL spectrum such as turnaround time have been largely overlooked in published studies.

Of Malawi’s 28 districts, just seven have molecular laboratories capable of conducting viral load testing. The remaining 21 districts refer specimens to those districts with molecular testing laboratories. Our results indicated that districts without a molecular laboratory were associated with longer pretest phase turnaround time, suggesting that efficiency of specimen transfer to centralized testing sites may be one factor driving longer turnaround time in Malawi. In particular, the association with longer pretest phase turnaround time points to specimen transport time and/or time spent waiting for transport as possible drivers of the association.

We also found that use of DBS for VL testing has significantly higher odds of longer test phase turnaround time compared to plasma. While DBS typically travels further than plasma on its way to testing labs in Malawi, the association with test phase turnaround time, which measures time elapsed between receipt at the laboratory and testing, links DBS to more time spent in the laboratory. This association suggests that there are factors that delay testing of DBS specimens. Those factors may include technicians untrained in DBS preparation, bottlenecks related the DBS sorting and specimen rejection process, or preference for plasma due to pressure from nearby clinics, ease of preparation or limited cold-storage.

While centralization of testing and use of DBS increases access to VL monitoring, it is nevertheless susceptible to inconsistent specimen transport networks, staff shortages, weather, holidays, reagent stock-outs, equipment problems and administrative delays. Several of these factors have been noted as barriers to VL scale-up [19, 20] and many have also been documented in perception and feasibility studies [11, 21]. Whether or not these factors affect VL testing and thus turnaround time is largely dependent on where and when the specimen was collected and where and when the testing occurred.

Our data indicated that certain testing labs had much stronger associations with longer test phase turnaround time than others, suggesting that factors such as those mentioned previously play a role in laboratory to laboratory variation. Factors related to staffing, supplies or administration may be responsible for the differences between labs, but it is difficult to know without intimate knowledge of the lab. Our models also indicated that certain specimen collection months had higher odds of longer pretest phase turnaround time and certain receipt months had higher odds of longer test phase turnaround time. The collection months of April, May, November and December of 2015, for example, stood out with increased odds of longer pretest phase turnaround time, suggesting that issues related to sample transport such as poor weather or a fuel shortages may have been present during those months. Similarly, specimens received during November and December 2015 had substantially higher odds of longer test phase turnaround time, which may be linked may to reagent stock-outs or staff leave due to holidays.

While our results do not pinpoint the exact causes of longer turnaround time, they reveal the scope of the problem, inform strategies for improvement, and provide baseline data to assess future improvement efforts. Perhaps the clearest strategies to shorten turnaround time are to increase efficiencies and improve quality management systems (QMS) across the VL spectrum. Implementing these strategies in LMICs, however, is challenging, particularly implementation of comprehensive QMS. Thus, use of focused, data-driven investigations into laboratory quality issues in lieu of comprehensive QMS may be a more feasible option. The key to such investigations are data collection systems reminiscent of Malawi’s LIMS, which are akin to surveillance systems long utilized in public health to detect and respond to disease outbreaks. In the case of LIMS, though, the disease is sub-standard laboratory quality, such as long turnaround time, and the response is a targeted investigation to identify and address causes responsible for that sub-standard quality. Future efforts in Malawi and elsewhere should treat such systems, many of which are already in existence, as they would traditional surveillance systems; that is, the data being collected should be regularly monitored and the system collecting the data should be periodically evaluated. Optimized use of these systems will enable LMICs to efficiently utilize resources to improve laboratory quality and ensure that health outcomes are not compromised.

The implications of shortened turnaround time are not limited to improved health outcomes. Other aspects of the VL spectrum, such as demand for and quality of VL testing are essential to reaching the 90-90-90 goals [1] and may also be positively affected by shortened turnaround time. A 1989 study conducted in an American hospital alluded to a phenomenon in which the number and type of tests ordered within the hospital were influenced by turnaround time, and suggested that decentralization of testing could shorten turnaround time [22]. While the setting is vastly different, that study is echoed by recent anecdotal reports from Malawi that suggest demand for VL testing and movement toward decentralized testing are influenced by turnaround time. Specifically, longer turnaround times may be decreasing demand for VL testing and driving decentralization via adoption of point-of-care (POC) technology, which, though promising, raises concerns about quality [23, 24]. These and other turnaround time-affected aspects lend additional urgency to turnaround time improvement efforts.

Key strengths of this study include the nationally-representative data and the focus on factors that affect turnaround time for VL testing not only in Malawi, but in many LMICs. These data contribute to a better understanding of VL turnaround time and provide evidence that supports the need for a stronger, more efficient VL spectrum. Limitations include the access to data only for afferent turnaround time, which limits our ability to connect findings to patient indicators such as adherence counseling, treatment switches, and patient notification of VL results. Additionally, an adequate duration for HIV VL testing turnaround time has not been identified by either the Malawi Ministry of Health or international health bodies such as the WHO. This lack of a proverbial ‘measuring stick’ for turnaround time limits our ability to judge the adequacy of the turnaround times observed in Malawi. Finally, there were a substantial number of VL specimens with missing and/or implausible date combinations, which were omitted from turnaround time calculations.

## Conclusions

Longer afferent turnaround times for VL testing were observed in Malawi and appeared to be driven in a phase-specific manner by factors such as the presence of a molecular lab in the collection district, the type of specimen collected, the month that the specimen was collected, and the laboratory where the test was run. Given turnaround time’s impact on the VL spectrum, addressing the specific causes of longer turnaround time nested within these factors is critically important. Strengthening the VL spectrum via increasing efficiencies, improved QMS, and capacity to conduct data-driven investigations into laboratory quality issues are initiatives that should be considered essential to reaching the 90-90-90 targets and controlling the HIV epidemic.
